# Hemiplegia Laryngis in the Horse (Roaring)1Paper read before the meeting of the Society for the Advancement of Veterinary Science, at Utrecht, September 22, 1900.

**Published:** 1901-06

**Authors:** M. W. J. P. Thomassen

**Affiliations:** Instructor at the Government Veterinary School at Utrecht


### HEMIPLEGIA LARYNGIS IN THE HORSE (ROARING).[1]

BY PROF. M. W. J. P. THOMASSEN,
INSTRUCTOR AT THE GOVERNMENT VETERINARY SCHOOL AT UTRECHT.

*Translated from the Tijdschrift voor veeartsenijkunde,*

BY L. VAN ES, M.D., V.S.,
MEDICAL DEPARTMENT UNIVERSITY OF ALABAMA, MOBILE, ALA.

(Concluded from page 283.)

*Pathological-anatomical Changes.* The degenerated muscles are paler than normal, yellowish-brown, and totally discolored in the worst forms. They soon decrease in volume, which is especially evident by comparison with the muscle of the other side, preferably after incision. These changes are to be seen the plainest in the posticus. In general, the left normal posticus is less developed

[1] Paper read before the meeting of the Society for the Advancement of Veterinary Science, at Utrecht, September 22, 1900.

than the right. Therefore, it is well to always experiment on the right side. Microscopical examination shows that the muscle bundles are reduced to one-half or one-third of their normal volume. The cross-striped appearance is soon lost. The interstitial connective tissue soon gets in a condition of proliferation, not only between the bundles, but also between the muscle fibres it is much increased, so that the primary bundles may be pressed far apart. The connective tissue nuclei may be considerably increased, so that they completely fill the interstitial space between the muscle fibres.

*Resection of the Superior Laryngeal Nerve.* Although the superior laryngeal nerve in the horse is put down as a sensory nerve exclusively notwithstanding the anastomoses with branches of the recurrent nerve, and in this animal this does not provide any of the laryngeal muscles with motor fibres; according to some the muscles undergo considerable changes after resection of this nerve. It has even been imagined that motor disturbances were already seen immeditaely after the operation.

Möller[1] is the first to report a resection of the superior laryngeal in two horses. The first experiment animal was killed six weeks after the operation, after it had been established first that no laryngeal stenosis existed. All muscles on the side concerned were found to be in a condition of atrophy. They were lighter in color and reduced in volume. The second horse was killed four and one-half months after the resection. In this horse, too, a marked muscular atrophy was observed, although here, also, respiratory trouble was absent.

Based upon these results, Möller concludes that the nerve contains trophic fibres. This experiment should furthermore prove that partial muscular atrophy is not sufficient to cause roaring.

Following Möller, Exner,[2] of Vienna, convinced himself, first, that by stimulation of the superior laryngeal nerve in the living animal not a trace of muscular contraction occurs. After this a piece was taken from the nerve. Before the operation the movement of the larynx was watched by means of the laryngoscope and found to be normal on both sides. Immediately after the operation the left vocal cord and the arytenoid cartilage stood still. The vocal cord had assumed the cadaver position and persistently remained immovable in this posture.

Pineles,[3] his pupil, plainly found degeneration phenomena in

[1] Das Kehlkopfpfeifen des Pferdes, 1888.
[2] Centralblatt für Physiologie, 1889.
[3] Pflüger's Archiv, 1891.

the muscles, which, according to him, differ markedly from the changes to be observed after resection of the recurrent. Pineles examined the larynx forty-five days after resection of the superior laryngeal. After hardening in alcohol longitudinal and transverse sections, stained in hæmatoxylin and eosin, were examined. The muscles, including the crico-thyroideus, were, even in fresh conditions, plainly pale red to yellow.

The microscopical changes have great similarity with those seen in dystrophia muscularis progressiva (Erb). Beside atrophy we often have hypertrophy of the muscle fibres. The latter precedes the first.

In general, the muscle bundles have become thinner. Beside this striking process there was swelling to be observed in other bundles; this became less by increasing degeneration. In the thyro-arytenoideus superior it is entirely absent. Beside the increase in volume the appearance of the primary bundles was normal, while the atrophied ones had nearly entirely lost their cross-striped appearance, but, on the contrary, were more striped longitudinally. In another horse Exner saw the same changes. Also the crico-thyroideus was markedly atrophic. Exner does not accept a trophic disturbance, but regards the case as an inactivity atrophy, the loss of sensation being the cause of the apparent motor disturbance.

Other experiments at the Berlin Veterinary School by Breisacher and Gützlaff gave results[1] contrary to the preceding. In two horses a piece of the superior laryngeal was taken in the vicinity of the thyroid cartilage. The animals remained alive three and one-quarter and three and three-quarter months after the operation. At the autopsy all laryngeal muscles were found to be normal; they even did not show discoloration. Also in dogs and rabbits they did not see, after resection of the nerve, degeneration of the muscles supplied by the recurrent. From this the results of the experiments of Möller and Exner seem to be doubtful.

Some years later Exner did not get the same result again.[2] In two horses operated upon symptoms of paralysis failed to appear to begin with, and after death there was no trace of muscle degeneration to be found. His explanation of the case, that an exchange of function between the superior and inferior laryngeal nerves could exist, we regard as far-fetched and improbable.

[1] Centralblatt für die medicinischen Wissenschaften, 1889.

[2] Centralblatt für Physiologie, 1891, p. 589.

Munk[1] also does not attach value to the results of the first two experimenters, on the strength of the experiments of Breisacher. He accounts for the paralysis and muscular atrophy by an accidental recurrent paralysis and with so much the more reason as both operated on the left side. In numbers of horses there might be, according to him, an incipient muscular atrophy on the left side, in which during life respiratory disturbances have been absent.

In a one-and-a-half-year-old horse with paresis of the hind-quarters I severed the right nerve close to the thyroid cartilage. After that the patient remained alive fully two months. In so far as the animal could be caused to move on account of the paralysis there was nothing to be detected of stridor. Two days before the killing the animal was placed in the dorsal position and the larynx was laid open. It was plain that the right arytenoid cartilage remained unmoved in the cadaver position while the left made considerable movement. I show you here the larynx of the horse in question. Macroscopically there are, as you see, no appreciable changes to be observed. The muscles of the right half compared with those of the left differ from them neither in color nor in volume. On this preparation all muscles appear somewhat pale, perhaps a consequence of the young age of the animal, and perhaps of the manner of treating the preparation, which may have remained too long in alcohol. For the microscopical examination two small pieces have been taken from the two posterior arytenoid muscles; as much as possible from the same location, hardened, and after that sectioned. The sections were stained with hæmatoxylin and eosin, and showed, compared with one another, little difference. At first one would have said that at some places in sections of the right muscle there existed increase of connective tissue nuclei and that some muscle bundles were somewhat smaller than those of the other side, but the difference was not of such a nature that one could attach value to it.

There remains, thus, nothing but the lesser motility which probably is the result of the anæsthesia of the mucosa. At any rate, we learn from these experiments that a functional disturbance of the superior laryngeal nerve never can be regarded as the cause of roaring in the horse.

That the results after resection of the superior laryngeal nerve can be so different could for a single case be explained from the following fact: On this larynx (Fig. 4) we see at a distance of

[1] Archiv für Thierheilkunde, B. xviii.

7 cm. from the thyroid cartilage the nerve split into two equally large branches, of which one enters along the usual opening and the other one 2 cm. lower along the posterior surface of the thyroid cartilage. The lower probably ramifies itself more in the muscles than the upper. If in this horse a neurectomy had taken place close to the opening—the usual place—the damaging influence on the muscles, of whatever nature this may be, would have remained absent. It appears that this anomaly occurs often. A division just above the opening, whereby both branches entered through the opening, I observed myself.

*Electrical Stimulation of the Laryngeal Nerves.* The results of direct stimulation of the different muscles have already been mentioned above. The two nervi laryngeus superior et inferior or recurrent were stimulated partly immediately post-mortem, partly *intra vitam.* Currents of varying intensity applied to the superior laryngeal caused no muscle reaction. Dilatation occurred after irritation of the recurrent nerve with a weak current by means of a Du Bois-Reymond apparatus. This was also the case in the living animal. As soon as the coil distance was placed at 28 cm. constrictors resulted, even so intense that in the cast horse after unilateral irritation a tolerably marked stridor could be heard, which immediately ceased as soon as the current was weakened by moving the coil. This phenomena repeated itself every time by replacement at the distance above mentioned. Consequently a weak current causes abduction (dilatation) and a stronger one adduction (constrictor) of the larynx.

In a three-year-old horse, which, as a result of left recurrent paralysis, suffered in a severe degree from roaring no laryngeal dyspnœa occurred in the recumbent animal after application of an electric current. The nerve was pulled outward and rested on two small glass rods. There was only seen violent spasms of the sternomaxillaris and levator humeri muscles. At first I thought of the results of a direct connection with the tracts of the spinal accessory nerve, but after severing the recurrent the spasms did not occur during irritation of the central extremity. The cause must thus be looked for in the peripheral portion. The spasms can be explained as the result of so-called "Stromschleifern"—that is, the diversion of the current of the irritated nerve to certain nerves situated in the vicinity. In this case there is an opportunity for this, as a branch of the accessory nerve crosses over the recurrent just under the larynx.

Some imagine that they have seen movement in the vocal cords of the opposite side by irritation of the central stump of the cut recurrent. This, however, has been contradicted by others on the ground that the recurrent did not possess centripetal tracts.

*Clinical Recurrent Paralysis.* Degeneration and atrophy of the laryngeal muscles do not show themselves as regularly in spontaneous paralysis as after resection of the recurrent nerve. Sometimes, as we stated above, the trophic disturbance of the constrictors is the most prominent, but, as a rule, this is the case with the dilator posticus; in other cases the degeneration keeps pace in all muscles. In the horse we do not so often see an isolated posticus paralysis as is the case in man, where an isolated degeneration of this muscle may exist for years without the adductors and the crico-thyroid having undergone the least change. Semon saw a case of posticus paralysis which persisted for months as the result of an aortic aneurism. In tabes this is by no means rare. In other cases it was seen that when recovery took place, in total recurrent paralysis of man, the adductors recover much sooner than the abductors, so that we have the picture of recurrent paralysis changed to that of a posticus paralysis. In 1892 Dr. Burger described thirteen cases of this sort. Sometimes the posticus paralysis existed by itself. Even in complicated central nervous paralysis the paralysis of this dilator, which probably was too much degenerated to be able to recover, sometimes persists by itself after recovery of the other muscles. This phenomena was at first declared to be the result of a biological inferiority of the posticus muscle as compared with the adductors. This muscle also dies off sooner and loses its electrical irritability earlier than the other muscles of the larynx (Jeanselme and Lermoyez). I have many times been able to convince myself of the truth of this on the larynx of the horse. We have already pointed to the greater irritability of these muscles. The consequence of this is that a weak current acting on the nerve causes dilatation and a strong current constriction (Gützner and Simanowski). The same phenomena presents itself on the pinchers of the lobster, which are opened by weak stimuli and closed by strong ones (Richet and Luchsinger).

Rosenbach has compared the adductors and abductors of the larynx with flexors and extensors of the extremities. It is known that flexors have a greater power of resistance and thus become paralyzed later than extensors. The extensor muscles lose their irritability earlier than the flexors. Gerhardt saw when he gently ligated the ischiatic nerve with a woollen thread soaked in turpentine, degeneration reaction occur, sometimes exclusively in the muscles of the peroneus regions, or at least in those long before in the other muscles. It has been tried to explain in various other ways why in organic paralysis the dilators degenerate first. Mackenzie thought that the dilators by their superficial location were more exposed to harmful influences than the constrictors. Others maintain that the constrictors have a special additional supply from the superior laryngeal (Cohen Verveart). If this were the case it could not be explained why in man and horse, after an isolated posticus paralysis at first, a total recurrent paralysis can gradually develop.

After the posticus muscle the internus comes next. Then follows, according to Semon, the transversus, and, finally, the lateralis. In the horse the atrophy of the transversus often exceeds that of the laterally placed muscle.

Cagney believes that the dilators perish sooner, because they are not represented in the cerebrum. Beside, in the greater irritability and consequently greater vulnerability of the extensors the explanation of the pathological phenomena above mentioned has also been looked for in an anatomical property of the recurrent nerve.

As was already predicted by Semon, the isolated course within the recurrent of the nerve tracts is destined for the different muscle groups of the larynx, and the dilator and constrictor fibres were later separately shown by Russell. Russell has divided the recurrent into three bundles, of which one acts dilating and a second constricting. He calls the third indifferently.

Exposed to the air, the dilating tracts lose their conductive power earlier than those by which the constrictors are innervated. Also, in clinical paralysis of the posticus the involved recurrent is found to be partially degenerated. It often contains nerve fibres still intact.

Koschier[1] found in one case the branch that goes to the posticus degenerated only. Onodi[2] describes a similar case. I had occasion frequently to observe this phenomenon by a microscopical examination of the recurrent nerve. About this later.

The dilator tracks in the recurrent also lose their electrical irritability and conductive power earlier than those destined for the constrictors of the glottis. From this it may be concluded that

[1] Wiener klinische Wochenschrift, 1897.
[2] Die Innervation des Kehlkopfes, 1895.

the lesser power of resistance of the dilator apparatus of the glottis does not limit itself to the muscles, but also originates in the nerve.

Burger,[1] on the strength of many examinations, comes to the following conclusions: There exists a physiological difference between the antagonistic muscle groups of the larynx, and this not only holds good for the muscles, but also for the nerves concerned. The dilators of the glottis are more irritable than the constrictors, but also perish sooner. From this comes the predisposition of the dilators for pathological changes.

In this preparation we see the recurrent divided in two parts. The division took place without any destruction of nerve fibres.

What will be the consequence of an independent paralysis of the dilators? In other words, what influence will the paralysis of the posticus have on the respiration? After extirpation of the posticus the degree of constriction of the glottis is much more marked in median portions than after recurrent resection, whereby we have, also, paralysis of the adductors. If after severing the posticus muscle a recurrent resection also takes place then is this immediately followed by a considerable dilatation of the *rima glottidis* (Schmidt,[2] Grabower, Klemperer, etc.). This phenomenon can be compared with what we see in unilateral paralysis of paired nerves, among others in the so frequent inferior facialis paralysis of the horse. We see, then, asymmetry of the face—that is to say, that the nose and lips are drawn toward the normal side. Similar, also, in paralysis of an antagonist. For example, in abducens paralysis the eye turns under the influence of the rectus internus toward the nose; in oculomotor paralysis toward the external canthus.

The normal muscles (antagonists) are in a condition of continued shortening, known under the name of secondary contraction.

The median position in posticus paralysis proves the greater functional power of the adductors, and is perfectly equal to what happens in facialis or abducens paralysis.

The secondary contraction especially relates to the muscles whose points of attachment, after paralysis of the posticus, have been brought closer together in consequence of their own tone, and those in the first place are the lateralis and the transversus less the thyro-arytenoid muscles, and the crico-thyroideus not at all.

Here, also, like everywhere in the body, the antagonists of the

[1] Zur Stimmbandstellung nach Recurrensdurchscheidung und zur Fraye der Posticuslähmung. Berlin, 1899.

[2] Die Laryngoskopie an Thieren. Tübingen, 1873.

paralyzed muscle group get into a condition of constant contraction, because at the cessation of their physiological activity the return to their normal condition of rest cannot be actively enhanced by the action of the paralyzed muscles under consideration.

The action of the elastic membrane alone is not sufficient to return the contracted constrictors as at rest. The paralyzed vocal cord and arytenoid cartilage are more and more drawn toward the median line, and finally, as it were, remain fixed in this position.

Of practical value it may be concluded from this that probably in the horse also an isolated posticus paralysis often occurs, so that by contraction of the constrictors the stenosis laryngis is more prominent than in the cases where all muscles degenerate in the equal manner. It is thus possible that there is contraction of the constrictors, especially in part of the cases of roaring of a recent origin, where we may observe an intense stridor. After resection of the recurrent nerve we did not observe the most severe degree of roaring.

This probably gives us the explanation of the observation reported by Möller,[1] that sometimes by the total immobility of the arytenoid cartilage a slight stridor was observed, and in other cases where still some mobility existed an intense inspiration sound. The practical conclusion for the therapy to be made from what has been mentioned would be, that in the most severe cases of nervous roaring in the horse a resection of the recurrent nerve is indicated. I have had occasion to experiment already in this direction, but did not obtain the desired result. The experimental investigation, furthermore, teaches that the age has a marked influence on the degree of stenosis laryngis, and, consequently, also, on the intensity of the roaring. The younger the individual the more violent the stridor after recurrent resection; the same, also, in spontaneous or clinical paralysis. Although frequently observed also in horses which became roarers at a far advanced age, the highest degree of stridor where nothing but a hemiplegia was the cause of the stenosis.

In a couple of cases of horses above twelve years old, after a unilateral resection of the nerve, roaring was so insignificant that it could hardly be noticed when led in a trot, and immediately ceased as soon as the animal stopped. The explanation of this fact is to be found simply in the greater rigidity of the tissues in animals of a more advanced age, whereby the arytenoid cartilage

[1] Das Kehlkopfpfeifen der Pferde. Stuttgart, 1888.

remains better fixed in the lateral position. Because what happens in recurrent paralysis? The involved arytenoid cartilage hangs motionless in the lumen of the larynx. Sometimes a short, jerky movement upward and sideward is still seen during beginning inspiration. This is brought about by the influence of the normal even hypertrophied crico-thyroid muscle, which brings the bezel of the cricoid cartilage upward and backward and hereby elevates somewhat the arytenoid cartilage, which is attached to the cricoid cartilage. But by the entering air column the arytenoid is directly brought back to its former position, even dependent on the force of the inspiration current, pushed still further in the lumen of the larynx in a median direction; at the same time the ventriculus becomes inflated.

In the long run the cartilage, as it were, grows fast in this position. The more relaxed the soft parts now are the further will the arytenoid cartilage be displaced inward and downward.

Dieckerhoff maintains that this displacement of the left arytenoid cartilage will become greater by hypertrophy and increased function of the right arytenoid muscle. Perhaps it is possible that in far advanced cases the cartilage mentioned is maintained more or less in the inspiration position by the fibrous tissue like totally degenerated muscles, of which I can show you a couple of specimens derived from even very young animals. The stridor originates principally by the air striking during inspiration against the upper edge of the arytenoid cartilage medially displaced. The position of the vocal cord contributes little to this.

The spontaneous recurrent paralysis in the horse nearly always occurs on the left side. Investigators of repute pretend never to have observed an isolated paralysis of the right half. K. Günther says: "Paralysis of the right recurrent by itself has not been demonstrated."[1] The opinion of Dieckerhoff is: "The muscular atrophy on the right side of the larynx has been found by Prinz (*Jahresbericht*, Dresden, 1837–1838) at the autopsy of a horse. Gerlack reports on a preparation to be found in the collection at Berlin, mounted by Gurlt, on which the laryngeal muscles of the right side are wasted and the right recurrent nerve shows a cicatrized injury. I have not been able to find in the literature other cases of exclusively right-sided recurrent paralysis."[2]

In this respect I was more fortunate. I have the opportunity to show you a very interesting preparation. You see here (Fig. 5)

[1] Studien über das Kehlkopfpfeifen der Pferde, etc. Karlsruhe, 1896.
[2] Gerichtliche Thierheilkunde, 2te Auflage, p. 366.

the larynx of a two-year old horse, treated by me for a nasal dyspnœa, the result of a chronic serous catarrh. The atrophy of the muscles of the right half, except the crico-thyroideus, is uncommonly well marked. In the place of the muscle substance only a small sinewy strip is to be found. Although this horse trotted the distance of about one hundred metres many times in succession, there was nothing to be heard of a laryngeal stenosis sound. Only in neighing was there a high degree of hoarseness, almost total aphonia, to be observed. This led us to examine the larynx more closely after death. That in this horse no stridor laryngis was noticed is caused only by the lesser volume of the inspiratory air column as the result of the nasal stenosis.

*Paralysis of the Adductors.* We made, currently, mention of the isolated paralysis of the constrictors. I will show you a larynx in which this change is to be observed in *optima forma.* In human medicine it is put down, as we already stated, under the name of functionary paralysis, of which the cause should be exclusively situated in the cerebrum. Semon says that no preparation is found in which the dilators are more degenerated or atrophied than the constrictors, as a result of an organic progressive disease of the laryngeal motor nerves; on the contrary, cases in which posticus atrophy occurs by itself are many fold. Schnitzler and Solis-Cohen do not agree with Semon on this subject. The only disturbance by which it shows itself during life is hoarseness on total aphonia, and in the horse, perhaps, by a characteristic cough with open glottis, by which this becomes abnormally stretched. Contrary to what Semon maintains in the case of man, in the horse the atrophy of the constrictors often has the start on that of the dilators, or it occurs isolated. In the latter case troubles in respiration are absent. This hemiparesis has, in the animal species mentioned, a purely peripheral character, and has no connection with an affection of the central nervous system, as is evident from the consequent posticus involvement. Posticus paralysis causes no change in the voice. Semon speaks of a lyric tenor with the left vocal cord in the median position, as the result of a recurrent paralysis, remaining after syphilis. Notwithstanding the total immobility of the vocal cord the voice was perfectly normal, the lung tone as well as the falsetto.

The internus, here called inferior, is the most important muscle for phonation, and in man is therefore named the sing-muscle. Its paralysis causes in animals hoarseness (aphonia). This larynx (Figs. 6, 7, and 8) comes from a three-year-old stallion, in which hoarseness only was observed. Stridor had never been heard. As you see, the left half of the transversus, the left lateralis, and the thyro-arytenoideus inferior are especially atrophied, the latter of which has almost entirely disappeared. The superior is still the last atrophied, although the transversus, in which this muscle passes, is degenerated in a great measure.

The posticus is about normal, so that we may suppose that the dilatation of the larynx during inspiration took place in a sufficient manner—a reason why no stenosis sound was observed.

In young stallions which have served and often also have not served, even in cryptorchids, we ascertained during neighing frequently disturbance in phonation. It appeared on closer examination that those animals were yet entirely free from roaring; in other cases this ailment existed only in a very slight degree, but, although slowly, it became worse. In the so-much-talked-about stallion Emi this was the case; at the time I considered it an alteration of the mucous membrane to account for the hoarseness; more so as the mucosa in front of the entrance to the larynx showed traces of a chronic affection, coupled with hypersecretion. From all this follows that the differentiation accepted in human medicine probably is not to be applied to the disturbance in question in the horse.

Before all, I hesitate to regard this ailment, also in isolated paralysis of the constrictors, as of cerebral origin, and principally because it is always unilateral, and the posticus paralysis frequently appears after a shorter or longer period and shows itself by disturbance in respiration. Furthermore, in the cases referred to, other nervous disturbances along with the laryngeal trouble are absent. We must consider this unsoundness in the horse to be of peripheral origin, of which the genesis is probably not identical in this with the common recurrent paralysis, whereby the posticus atrophies first.

Finally, a single word more about the clinical recurrent paralysis in man, of which, according to the rule of Semon, three stages are recognized, which, briefly, amount to the following:

1. Paralysis of the posticus. The vocal cords are removed 2 mm. from the median line. During phonation and forced expiration they approach the median line.

2. Paralysis of the posticus with contraction of the antagonists. The vocal cords have completely assumed the median position.

3. Paralysis of all laryngeal muscles, innervated by the recurrent nerve. The vocal cords are in the cadaver position.

Before closing I have to make a request of you. In order to bring the second part of my investigation to a good termination I ask the support of all of you and beg you to send me preparations —that is to say, the larynx—preferably of animals known to you as roarers. The larynx with a few rings of the trachea should be cut out as freely as possible, so that the nerves will be partly saved and the preparation should be placed for one day in a 10 per cent. formalin solution.

---

DIPHTHERIA IN THE HORSE. Dr. Fraser (*Lancet*), of the laboratory of Portsmouth, examined the nasal mucus of a horse in order to determine the source of infection of diphtheria in a child. The latter's father owned a horse affected with sublingual adenitis, obstruction of the larynx, and difficult respiration. Fraser found a bacillus entirely identical with that of diphtheria, both as to morphology and culture; it was pathogenic to the guinea-pig and produced a very powerful toxin. He concluded that it was the Klebs-Löffler bacillus, and that the horse suffered from diphtheria.

From a scientific point of view this may throw some light on the question of the origin of antitoxin in the horse. It is known, in fact, that quite often the normal serum of man and of a healthy horse, without immunization with the bacillus or its toxin, is capable of neutralizing to diphtheria toxin, and it has been demonstrated that, in the horse at least, the blood contains a veritable antitoxin.

Wasserman suggests that this antitoxin in man as well as in the horse may be produced by an attack of diphtheria which disappeared unnoticed.—*Rec. de Méd. Vét.*

DERMOID CYST ON THE OMENTUM. Petit found a multilocular dermoid cyst in the omentum of a horse succumbed to tetanus. It was oval and flattened 10 cm. in diameter, and excavated by several cavities. Some of the latter contained a product resembling honey in consistency, while in others it was putty-like; one of these cavities contained a bunch of hair matted together and attached to the wall of the sac.—*Rec. de Méd. Vét.*

